# Esophageal Doppler-guided fluid management decreases blood lactate levels in multiple-trauma patients: a randomized controlled trial

**DOI:** 10.1186/cc5703

**Published:** 2007-02-22

**Authors:** Ivan Chytra, Richard Pradl, Roman Bosman, Petr Pelnář, Eduard Kasal, Alexandra Židková

**Affiliations:** 1 Department of Anesthesia and Intensive Care Medicine, University Hospital, Alej svobody 80, Plzeň 30460, Czech Republic

## Abstract

**Introduction:**

Esophageal Doppler was confirmed as a useful non-invasive tool for management of fluid replacement in elective surgery. The aim of this study was to assess the effect of early optimization of intravascular volume using esophageal Doppler on blood lactate levels and organ dysfunction development in comparison with standard hemodynamic management in multiple-trauma patients.

**Methods:**

This was a randomized controlled trial. Multiple-trauma patients with blood loss of more than 2,000 ml admitted to the intensive care unit (ICU) were randomly assigned to the protocol group with esophageal Doppler monitoring and to the control group. Fluid resuscitation in the Doppler group was guided for the first 12 hours of ICU stay according to the protocol based on data obtained by esophageal Doppler, whereas control patients were managed conventionally. Blood lactate levels and organ dysfunction during ICU stay were evaluated.

**Results:**

Eighty patients were randomly assigned to Doppler and 82 patients to control treatment. The Doppler group received more intravenous colloid during the first 12 hours of ICU stay (1,667 ± 426 ml versus 682 ± 322 ml; *p *< 0.0001), and blood lactate levels in the Doppler group were lower after 12 and 24 hours of treatment than in the control group (2.92 ± 0.54 mmol/l versus 3.23 ± 0.54 mmol/l [*p *= 0.0003] and 1.99 ± 0.44 mmol/l versus 2.37 ± 0.58 mmol/l [*p *< 0.0001], respectively). No difference in organ dysfunction between the groups was found. Fewer patients in the Doppler group developed infectious complications (15 [18.8%] versus 28 [34.1%]; relative risk = 0.5491; 95% confidence interval = 0.3180 to 0.9482; *p *= 0.032). ICU stay in the Doppler group was reduced from a median of 8.5 days (interquartile range [IQR] 6 to16) to 7 days (IQR 6 to 11) (*p *= 0.031), and hospital stay was decreased from a median of 17.5 days (IQR 11 to 29) to 14 days (IQR 8.25 to 21) (*p *= 0.045). No significant difference in ICU and hospital mortalities between the groups was found.

**Conclusion:**

Optimization of intravascular volume using esophageal Doppler in multiple-trauma patients is associated with a decrease of blood lactate levels, a lower incidence of infectious complications, and a reduced duration of ICU and hospital stays.

## Introduction

Post-traumatic hemorrhage in multiple-trauma patients leads to hypovolemia, in which blood flow and consequently oxygen delivery to the tissues are decreased. Reduction of oxygen delivery and oxygen consumption to below a critical level produces ischemic metabolic insufficiency followed by increased generation of lactate [1-4]. Blood lactate levels are closely related to outcome in critically ill trauma patients [4-9], and failure of serum lactate levels to reach normal values within a specific time during critical illness could be even more closely related to survival than the initial level [10-15]. According to a systematic Medicine/Cochrane Library literature search, blood lactate level was shown to predict outcome in almost 3,000 multiple-trauma patients [4].

Fluid resuscitation of trauma patients has traditionally been guided by the normalization of vital signs such as blood pressure, heart rate, and urine output. However, blood pressure and heart rate remain relatively unchanged despite reduced blood flow to certain tissues and hence they are insensitive indicators of hypovolemia and hypoperfusion [14,16-18]. Occult hypoperfusion, defined as elevated blood lactate levels without signs of clinical shock, was associated with increased morbidity and mortality, and early correction is likely to improve the outcome [7,8,10,19].

The esophageal Doppler is a non-invasive technique for monitoring cardiac function in intensive care unit (ICU) patients. The technique and clinical use were first described in 1971 [20], subsequently refined by Singer and colleagues in 1989 [21], and recently have been successfully approved for optimizing fluid management perioperatively and in intensive care patients [22-29]. Unfortunately, the Doppler probe is not readily tolerated by conscious patients, restricting its use to patients who are sedated and ventilated. To our knowledge, no prospective study has been performed to assess the efficacy of the esophageal Doppler for optimization of fluid management in multiple-trauma patients in the immediate postoperative period.

The aim of this study was to examine the effect of esophageal Doppler-guided fluid management during the first 12 hours after ICU admission on blood lactate levels, organ dysfunction development, infectious complications, and length of ICU and hospital stays in comparison with standard hemodynamic management in multiple-trauma patients.

## Materials and methods

This was a randomized, controlled, single-center study conducted in the interdisciplinary ICU of a university teaching hospital. The study was approved by the Local Research Ethics Committee of University Hospital in Plzeň (Czech Republic). Because the protocol was approved and regarded as part of the routine practice and (due to the emergency clause) informed consent by the patients or the family was not required, an independent physician was designated to give the consent. However, subjects were informed at discharge that they had participated in this clinical study.

### Participants

Ventilated patients with multiple trauma and estimated blood loss of more than 2,000 ml admitted to the interdisciplinary ICU of our university teaching hospital from 2003 to 2005 were considered for inclusion in this study. We excluded patients younger than 18 years old, patients with traumatic brain injury requiring treatment of intracranial hypertension, and those with relative contraindications to the use of the esophageal Doppler probe, such as orofacial and esophageal injury or other known oropharyngeal and esophageal disease.

### Protocol

Primary outcome measures were blood lactate levels after 12 and 24 hours after ICU admission and organ dysfunction development during ICU stay. Secondary outcome measures were duration of ICU and hospital stays and the incidence of infectious complications during ICU stay.

**Figure 1:**
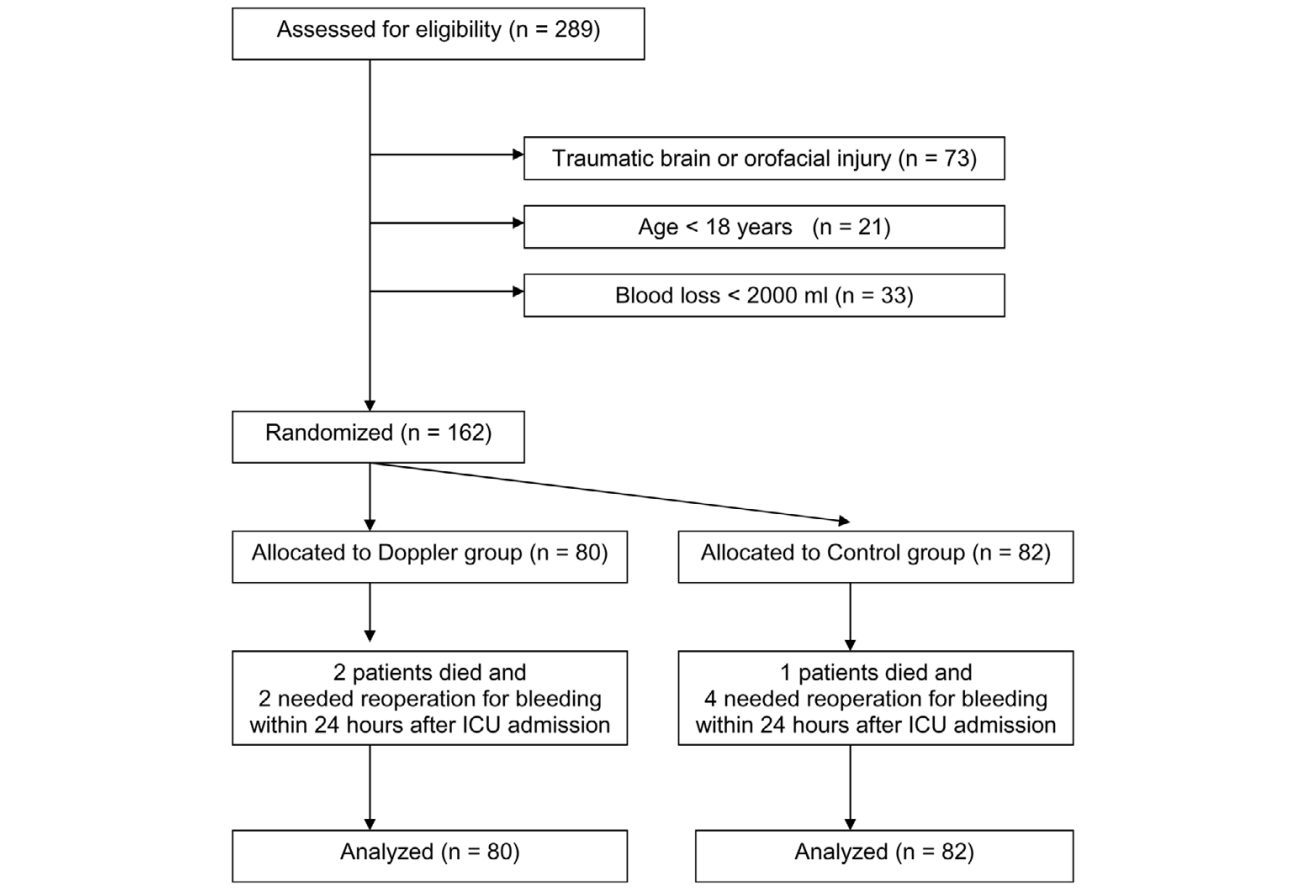
Flow of participants through the trial. ICU, intensive care unit.

Patients meeting inclusion criteria were randomly assigned to the protocol group (Doppler) or the control group according to the assigned admission number generated by the admission office of the university hospital (even: Doppler group, odd: control group). Randomization of the patients was performed by a member of the research team at the time of ICU admission. Data were analyzed on an intention-to-treat basis and included all patients who were randomly assigned (Figure 1).

Patients with multiple trauma were initially examined and treated in the emergency department of the university teaching hospital and after urgent surgery were admitted to the ICU. The amount of blood loss in the pre-study period was estimated by an emergency department physician and by an anesthesiologist taking care of the screened patient. Neither of them was a member of the research team. At the time of ICU admission and during the first 12 hours of ICU stay, all patients were mechanically ventilated (pressure-controlled ventilation) and received adequate continuous analgosedation (fentanyl + midazolam) to keep the Ramsay Scale score between 4 and 5 [30].

All patients were managed to maintain hemoglobin oxygen saturation (measured using pulse oximetry) above 95%, mean arterial pressure (MAP) above 65 mm Hg, heart rate below 100 bpm, urine output above 1 ml/kg per hour, temperature at 37°C, and hemoglobin above 85 g/l. Anemia and coagulation disorders in all patients were treated by administration of erythrocytes, platelets, and fresh frozen plasma (FFP) according to clinical and laboratory results. All patients received basic crystalloid infusion of 1.5 ml/kg per hour (Hartman's solution; B. Braun Melsungen AG, Melsungen, Germany). When necessary, norepinephrine was added to keep MAP above 65 mm Hg. Further plasma volume replacement in the Doppler and control groups was managed by colloid solutions administration of gelatine and hydroxyethylstarch in a 1:1 ratio (Gelofusine^®^; B. Braun Melsungen AG, and Voluven^®^; Fresenius Kabi AG, Bad Homburg, Germany). Fluid management in the control group was guided using the abovementioned routine cardiovascular monitoring and central venous pressure (CVP) measurement in order to keep CVP between 12 and 15 mm Hg.

**Figure 2:**
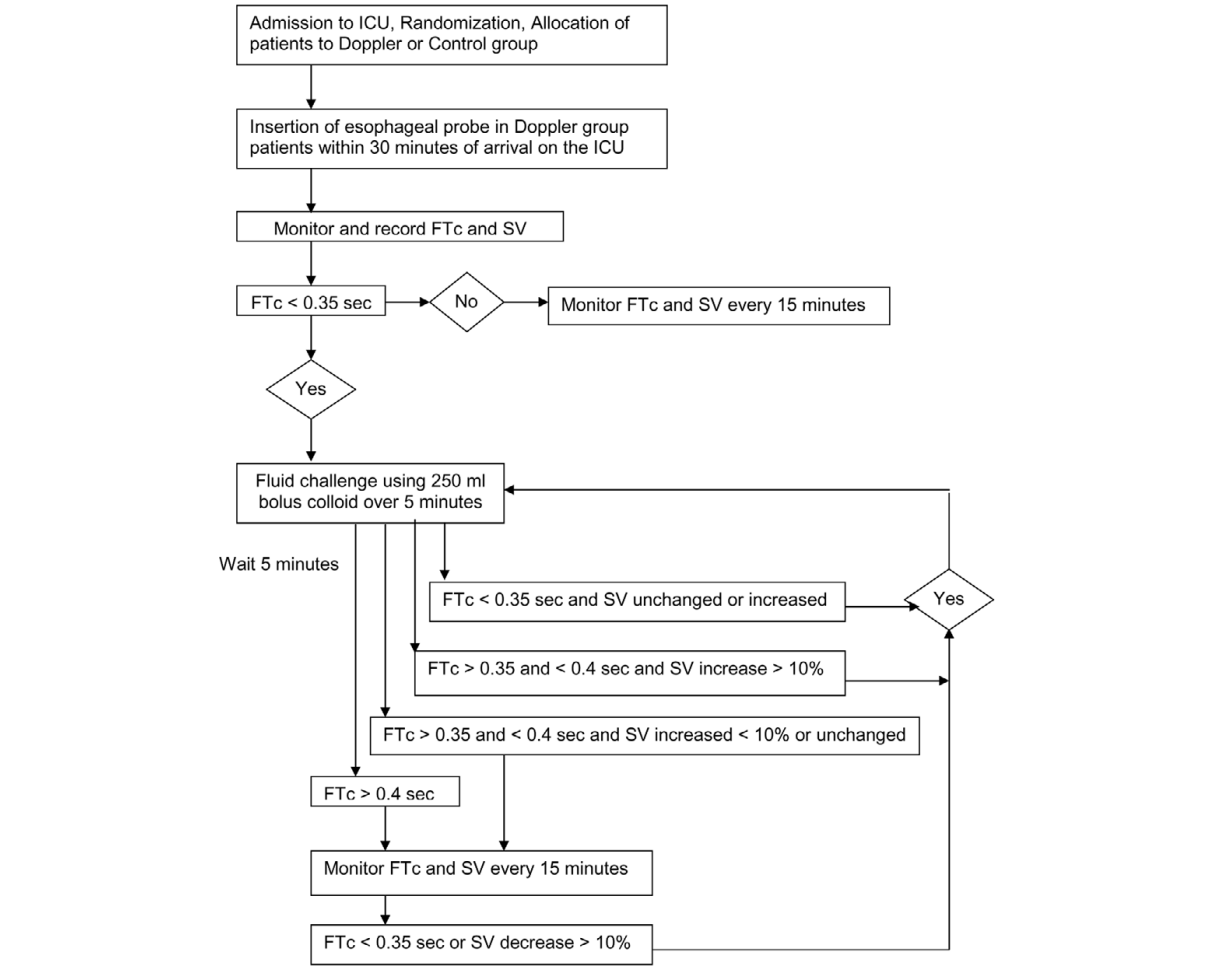
Fluid management algorithm in the Doppler group. FTc, corrected flow time; ICU, intensive care unit; SV, stroke volume.

In the Doppler-guided fluid replacement group, the esophageal 7-mm probe was placed into the lower esophagus through the mouth or nose to a depth of 35 to 40 cm from the dental row within 30 minutes after ICU admission. The probe was rotated as needed to obtain the best Doppler signal of blood flow in the midstream of the descending aorta. Correct placement was assumed when reproducible, sharply defined waveforms appeared on the screen of the monitor and crisp sound was heard through the loudspeaker. The algorithm for fluid replacement during the first 12 hours after ICU admission in the Doppler group was similar to that used by Sinclair and colleagues [23] (Figure 2). Corrected flow time (FTc) of less than 0.35 seconds was considered an indication of possible hypovolemia. Patients were given an initial bolus of colloid (250 ml) in a five minute period. If the stroke volume (SV) was either maintained or increased after the fluid challenge and FTc remained below 0.35 seconds, the bolus of colloid was repeated. If the FTc exceeded 0.35 seconds and the SV rose by more than 10%, the fluid challenge was repeated. If the FTc exceeded 0.35 seconds and SV was unchanged or rose by less than 10%, no further fluid was given until the FTc dropped below 0.35 seconds or SV fell by 10%. If the FTc rose above 0.40 seconds, no further fluid was given until the FTc dropped below 0.35 seconds or SV fell by 10%. Esophageal Doppler monitoring measurements were obtained using the Hemosonic 100 device (Arrow International, Inc., Reading, PA, USA), which enables continuous measurement of descending thoracic aorta blood velocity (Doppler transducer) and of aortic diameter (M-mode echo transducer). The technical details and relative merits of this technique have been reviewed elsewhere [31,32]. In the workplace where the study was implemented, the esophageal Doppler for hemodynamic monitoring has been used routinely for several years and all members of the research team were experienced in its use, and therefore measurement was performed by any of the clinical study investigators. This fluid protocol started immediately after probe placement and continued for 12 hours until the esophageal probe was removed. Following fluid management in both groups was guided in the same way as in the control group.

### Assessments

The following parameters were monitored during the study period: electrocardiograph, pulse oximetry, invasive arterial pressure, CVP, urine output, and (in the Doppler group) SV and FTc. Acute Physiology and Chronic Health Evaluation II (APACHE II) score and Injury Severity Score (ISS) were calculated after admission to the ICU. Sequential Organ Failure Assessment (SOFA) score was calculated daily, and the values at the time of ICU admission and the highest SOFA during ICU stay were assessed. MAP and CVP were evaluated at baseline and at the end of the 12-hour study period. Blood lactate levels were assessed at baseline and 12 and 24 hours after ICU admission. The normal value of blood lactate in our laboratory is less than 2.4 mmol/l. Rate and dose of norepinephrine, volume of administered crystalloids and colloids, and blood and FFP during the first 12 hours of the study period were assessed. Length of ICU and hospital stays, ICU and hospital mortalities, and incidence of infectious complications during ICU stay were evaluated. Diagnosis of infectious complications was established by non-research staff in accordance with predefined criteria [33]. Patients were followed up to hospital discharge.

### Statistical analysis

For the measure of primary outcome with reference to previous studies and our pilot data [13,15,34,35], we calculated a study size of 75 patients in each group to demonstrate the decrease of blood lactate levels by 0.6 mmol/l per 24 hours (standard deviation [SD] ± 1.3) in the Doppler group in comparison with the control group. For the measure of secondary outcome with reference to previous data [25], we calculated a sample size of 160 patients (80 in each group) by postulating a reduction in mean ICU stay from nine days in the control group to seven days in the protocol group (SD ± 4.5). Sample sizes were calculated for two-tailed tests allowing for a type I error of 5% and a type II error of 20%. The Kolmogorov-Smirnov test was used to check for normal distribution of data. Continuous normally distributed data were tested with the *t *test, and not normally distributed data were tested with the Mann-Whitney *U *test. Categorical data were tested with the Fisher exact test. Data are presented as means (SDs) where normally distributed and as medians (interquartile ranges) where not normally distributed. Relative risk is presented with 95% confidence intervals (CIs). A *p *value of less than 0.05 was considered statistically significant. Analysis was performed with MedCalc^® ^version 7.1.0.0 (Frank Schoonjans, MedCalc Software, Broekstraat 52, 9030 Mariakerke, Belgium).

## Results

A total of 162 patients were recruited between January 2004 and December 2005 (Figure 1). Eighty patients were randomly assigned to the Doppler group, and 82 patients to the control group. The groups were well matched for age, gender, SOFA score at the time of ICU admission, APACHE II score and ISS, and the type of injuries (Table 1). There were no differences between the Doppler and control groups in MAP, CVP, blood lactate level, and frequency and dose of norepinephrine administration at baseline (that is, at the time of ICU admission) (Table 2). After the 12-hour study period, blood lactate in Doppler group patients was lower (2.92 ± 0.54 mmol/l versus 3.23 ± 0.56 mmol/l; *p *= 0.0003) as were the dose of norepinephrine (0.093 ± 0.035 μg/kg per minute versus 0.169 ± 0.068 μg/kg per minute; *p *= 0.0009) and the rate of norepinephrine (18 patients [23%] versus 33 patients [40%]; relative risk = 0.56, 95% CI = 0.34 to 0.91; *p *= 0.018). We found no difference between the Doppler and control groups in MAP, but CVP in the Doppler group was higher (13.7 ± 1.8 mm Hg versus 12.1 ± 2.4 mm Hg; *p *< 0.0001). Patients in the Doppler group received a greater volume of colloid solutions (1,667 ± 426 ml versus 682 ± 322 ml; *p *< 0.0001) but similar volumes of blood, FFP, and crystalloid solution (Table 3). The difference of lactate level between the Doppler and control groups changed little after 24 hours of ICU stay (1.99 ± 0.44 mmol/l versus 2.37 ± 0.59 mmol/l; *p *< 0.0001). During ICU stay, no difference between the Doppler and control groups in the highest SOFA score was found (10 [7 to 12.75] versus 11 [7 to 14]; *p *= 0.17), but in the Doppler group fewer patients developed infectious complications (15 patients [18.8%] versus 28 patients [34.1%]; relative risk = 0.5491, 95% CI = 0.3180 to 0.9482; *p *= 0.032) (Table 4). The reduction of complications was associated with a reduction of median duration of ICU stay (7 days [6 to 11] versus 8.5 days [6 to 16]; *p *= 0.031) as well as with a reduction of median duration of hospital stay (14 days [8.25 to 21] versus 17.5 days [11 to 29]; *p *= 0.045) (Table 4). There was no significant difference in ICU and hospital mortalities (11 patients [13.8%] versus 16 patients [19.5%] [*p *= 0.40] and 13 patients [16.3%] versus 18 patients [22%] [*p *= 0.43], respectively) (Table 4). There were no complications related to esophageal Doppler ultrasonography.

**Table 1:** Baseline characteristics of patients in the Doppler and control groups

Characteristics	Doppler group	Control group
Number in group	80	82
Age in years	33 (26–57)	40 (26–50)
Male	73 (91%)	70 (85%)
APACHE II	20 (16–21)	18 (12–23)
Injury Severity Score	38.5 ± 10.5	36.4 ± 11.8
SOFA score at ICU admission	8 (7–10)	8 (6–11)
Type of injury		
Chest	42 (52%)	50 (61%)
Abdomen	52 (65%)	48 (58%)
Spine	24 (30%)	21 (26%)
Pelvis	36 (45%)	43 (52%)
Extremities	76 (95%)	76 (93%)

Values are presented as absolute (percentage) or mean ± standard deviation or median (interquartile range). APACHE II, Acute Physiology and Chronic Health Evaluation II; ICU, intensive care unit; SOFA, Sequential Organ Failure Assessment.

**Table 2:** Baseline parameters and therapeutic interventions

Parameters	Doppler group (*n *= 80)	Control group (*n *= 82)	*p*
Mean arterial pressure (mm Hg)	65 (60–71)	65 (55–75)	0.71
Central venous pressure (mm Hg)	7.1 ± 2.1	6.8 ± 2.1	0.34
Lactate (mmol/l)	4.2 (3.2–5.2)	3.9 (3.0–4.7)	0.08
Intervention			
Norepinephrine (patients)	58 (73%)	57 (70%)	0.73
Norepinephrine (μg/kg per minute)	0.23 (0.14–0.60)	0.21 (0.12–0.41)	0.25

Values are presented as absolute (percentage) or mean ± standard deviation or median (interquartile range).

**Table 3:** Therapeutic interventions and changes in parameters during the 12-hour study period

Parameters	Doppler group (*n *= 80)	Control group (*n *= 82)	*p*
Mean arterial pressure (mm Hg)	78 ± 6.5	79 ± 9.5	0.76
Central venous pressure (mm Hg)	13.7 ± 1.8	12.1 ± 2.4	< 0.0001
Lactate (mmol/l)	2.92 ± 0.54	3.23 ± 0.54	0.0003
Intervention			
Colloid (ml)	1,667 ± 426	682 ± 322	< 0.0001
Crystalloid (ml)	1,293 ± 300	1,334 ± 320	0.38
Blood (ml)	814 ± 228	833 ± 340	0.67
Fresh frozen plasma (ml)	742 (566–949)	750 (562–1,108)	0.69
Norepinephrine (patients)	18 (23%)	33 (40%)	0.018
Norepinephrine (μg/kg per minute)	0.093 ± 0.035	0.169 ± 0.068	0.0009

Values are presented as absolute (percentage) or mean ± standard deviation or median (interquartile range).

**Table 4:** Summary of outcomes after the 12-hour intervention period

Parameters	Doppler group (*n *= 80)	Control group (*n *= 82)	*p*
Lactate after 24 hours of ICU stay (mmol/l)	1.99 ± 0.44	2.37 ± 0.59	< 0.0001
The highest SOFA during ICU stay	10 (7–12.75)	11 (7–14)	0.17
Infectious complication			
Pneumonia	10	19	0.09
Central venous catheter	5	6	1.00
Abdominal	2	4	0.68
Urinary tract	3	2	1.00
Wound	2	5	0.44
Total infectious complications	22	36	0.033
Patients with infectious complications	15 (18.8%)	28 (34.1%)	0.032
Length of ICU stay in days	7 (6–11)	8.5 (6–16)	0.031
Length of hospital stay in days	14 (8.25–21)	17.5 (11–29)	0.045
ICU mortality	11 (13.8%)	16 (19.5%)	0.40
Hospital mortality	13 (16.3%)	18 (22%)	0.43

Values are presented as absolute (percentage) or mean ± standard deviation or median (interquartile range). ICU, intensive care unit; SOFA, Sequential Organ Failure Assessment.

## Discussion

Esophageal Doppler-guided fluid management in multiple-trauma patients decreased blood lactate levels, lowered the incidence of infectious complications, and reduced the length of ICU and hospital stays. Occult tissue hypoperfusion in trauma patients is relatively common and cannot be diagnosed and eliminated using traditional markers and resuscitation endpoints (blood pressure, heart rate, and urine output). Scalea and colleagues [16] found that up to 80% of critically ill patients who are normotensive and have adequate urine output may remain in a state of compensated shock. One of most commonly used markers in assessing occult tissue hypoperfusion in trauma patients is blood lactate. Several studies have shown that normalization of blood lactate levels within 24 hours of admission in hemodynamically stable trauma patients was associated with improved survival, less frequent infection rate, and organ dysfunction development [7,8,10-12]. Persistent elevated lactate levels 24 hours after admission significantly correlated with mortality [13]. Limited prospective data are available, but these indicate that rapid normalization of increased blood lactate levels is an important therapeutic goal in critically ill patients [19]. Adequate fluid resuscitation to increase cardiac output has been found to improve tissue oxygen delivery in patients with tissue hypoxia and remains the mainstay of therapy in these circumstances [36]. Esophageal Doppler flowmetry used to maximize intraoperative SV by repeated fluid challenges was associated with improved outcome and reductions in length of hospital stay after cardiac, orthopedic, or abdominal surgery [22-25,28]. Our data are in agreement with other studies [19,26,36] and support the statement that some beneficial effects might still be achieved from optimization of circulatory status in the immediate postoperative period.

Although decreased blood lactate levels in Doppler group patients during the first 12 and 24 hours of ICU stay indicate improved tissue perfusion and oxygenation, surprisingly we did not prove a significant difference between the Doppler and control groups in organ failure development during ICU stay. Presumably, the difference in tissue oxygen delivery in both groups was not significant enough to induce organ-function changes measurable by SOFA score. With reference to the 'golden hour' and the 'silver day' of trauma resuscitation [10,37], a partial explanation for this finding can be that despite the higher blood lactate levels in control group patients, oxygen delivery in both groups was sufficient to achieve normal lactate levels within 24 hours of ICU stay. However, other factors that could help to explain the uniformity in levels of organ dysfunction and mortality between the protocol and control groups (that is, amount of time elapsed between the injury and emergency department admission, duration of surgery, and amount of blood transfused before ICU admission) were not analyzed.

A relationship between the rapid normalization of blood lactate level and the lower rate of infectious complications in trauma patients was clearly demonstrated [7,8,10,36]. The blood lactate level in the control group after 12 and 24 hours of study was higher than in the Doppler group, and even though the blood lactate level after 24 hours of ICU stay in both groups reached the normal range, more patients in the control group developed infectious complications during ICU stay. Clinical studies support the notion that adequate fluid resuscitation may improve tissue oxygen tension and decrease the rate of complications [22,38]. Other studies have demonstrated that inadequate tissue perfusion measured with gastric tonometry is associated with adverse perioperative outcome [39,40]. Possibly, better tissue oxygenation results in improved tissue healing and decreased infection rate.

The use of esophageal Doppler for hemodynamic optimization based on administering fluids to achieve maximal left ventricular SV was associated with important reductions of ICU or hospital stay [22-26]. In the present study, esophageal Doppler-guided fluid management was associated with a 1.5-day median reduction in ICU stay and a 3.5-day median decrease in hospital stay. This suggests that optimization of circulatory status may also have financial implications and reduce the cost of care for multiple-trauma patients.

In a meta-analysis of hemodynamic optimization studies, Poeze and coworkers [41] showed that the use of strategies to optimize the hemodynamic condition perioperatively and during trauma significantly reduced mortality. In the present study, there was no statistical difference in ICU and hospital mortalities between the groups, and the study was not powered to show any difference in mortality. This would have required more than 700 patients.

There are some potential weaknesses in the design of our trial. It was of relatively small size, was not blinded, and was conducted in only one center. All patients in the control group received intravenous resuscitation guided by CVP measurement in order to keep CVP between 12 and 15 mmHg. However, because no reliable correlation between intravascular volume and absolute CVP measurement has been established, rather than apply an absolute target for CVP, dynamic changes of CVP to fluid challenge would likely provide a more reliable guide to fluid requirements [24,36]. Recruitment of patients was possible only when a member of the research team was available to administer the 12-hour study protocol. During the trial period, 539 multiple-trauma patients were admitted to the ICU and mortality was 22.4% (including deaths within 24 hours after ICU admission).

The application of the 12-hour study protocol in the Doppler group was time-consuming and would not have been feasible without the close cooperation of the nursing staff. The continuous presence of a clinician at bedside for a 12-hour period is not realistic and thus the fluid challenge according to the Doppler-guided protocol was given partly by trained nursing staff. Whenever the quality of the Doppler signal was altered (due mostly to a change of probe position resulting from nurse or patient movement), a member of the research team was called and the probe was restored to the proper position. In spite of adequate sedation, it was difficult to keep the Doppler probe in the right position for 12 hours without frequent adjustments. We suppose that the use of other relatively non-invasive devices that measure SV and cardiac output (for example, cardiac output measurement using partial carbon dioxide rebreathing, thoracic impedance, and technologies using arterial pressure waveform analysis) may be less demanding for postoperative fluid and hemodynamic optimization. Moreover, these methods do not require deep sedation, can be used for longer periods, and do not discriminate patients who are not suitable for esophageal Doppler monitoring.

## Conclusion

Optimization of intravascular volume using esophageal Doppler in multiple-trauma patients is associated with a decrease of blood lactate levels, a lower incidence of infectious complications, and a reduced duration of ICU and hospital stays. A large multicenter study should be performed to validate these findings and to demonstrate an effect on mortality.

## Key messages

• Optimization of intravascular volume using esophageal Doppler in multiple-trauma patients decreases blood lactate levels.

• Fluid resuscitation in multiple-trauma patients guided by esophageal Doppler is associated with a lower incidence of infectious complications and reduces ICU and hospital stays.

## Abbreviations

APACHE II = Acute Physiology and Chronic Health Evaluation II; CI = confidence interval; CVP = central venous pressure; FFP = fresh frozen plasma; FTc = flow time corrected; ICU = intensive care unit; ISS = Injury Severity Score; MAP = mean arterial pressure; SD = standard deviation; SOFA = Sequential Organ Failure Assessment; SV = stroke volume.

## Competing interests

The authors declare that they have no competing interests.

## Authors' contributions

IC and RP were responsible for study design and data analysis. All authors were responsible for administering the protocol, were involved in drafting the manuscript and approved the final version, and have full access to the data and take full responsibility for the integrity of the data.
